# Supplementary material for: Anti-friction gold-based stretchable electronics enabled by interfacial diffusion-induced cohesion
Source: Nat Commun. 2024 Feb 6;15:1116. doi: 10.1038/s41467-024-45393-x (PMC10847152; doi:10.1038/s41467-024-45393-x)
Supplement: Supplementary file 1 — Supplementary Information [file 41467_2024_45393_MOESM1_ESM.pdf]

Supplementary Information

Anti-friction gold-based stretchable electronics enabled by interfacial diffusion-induced cohesion

Jie Cao[1], Xusheng Liu[1,2], Jie Qiu[1], Zhifei Yue[1], Yang Li[1], Qian Xu[1,2], Yan Chen[1,2], Jiewen Chen[1], Hongfei Cheng[3], Guozhong Xing[4], Enming Song[5], Ming Wang[1,6], Qi Liu[1,2,6]*, and Ming Liu[1,6]*

[1]Frontier Institute of Chip and System, State Key Laboratory of Integrated Chips and Systems, Zhangjiang Fudan International Innovation Center, Fudan University, Shanghai 200433, China

[2]School of Microelectronics, Fudan University, Shanghai 200433, China

[3]School of Materials Science and Engineering, Tongji University, Shanghai 201804, China

[4]Key Laboratory of Microelectronic Devices & Integrated Technology, Institute of Microelectronics, University of the Chinese Academy of Sciences, Chinese Academy of Sciences, Beijing 100029, China

[5]Shanghai Frontiers Science Research Base of Intelligent Optoelectronics and Perception, Institute of Optoelectronics, State Key Laboratory of Integrated Chips and Systems, Fudan University, Shanghai 200433, China

[6]Shanghai Qi Zhi Institute, 41th Floor, AI Tower, No. 701 Yunjin Road, Xuhui District, Shanghai 200232, China

[*]Corresponding author. E-mail: wang_ming@fudan.edu.cn, qi_liu@fudan.edu.cn

Table of Contents:

I: Supplementary Figures and Captions

II: Supplementary Tables

III: Supplementary Note 1

VI: Supplementary References

I: Supplementary Figures and Captions

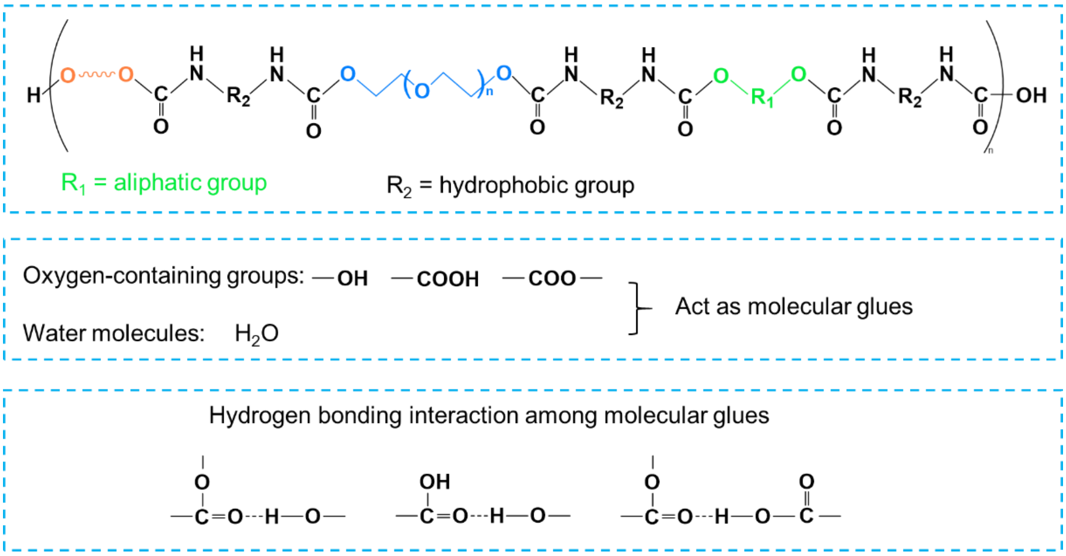

Supplementary Figure 1 Chemical structure of hydrophilic WPU. Schematic illustration of molecular structures of molecular glues and their hydrogen bonding interactions in the Au-RPU interface.

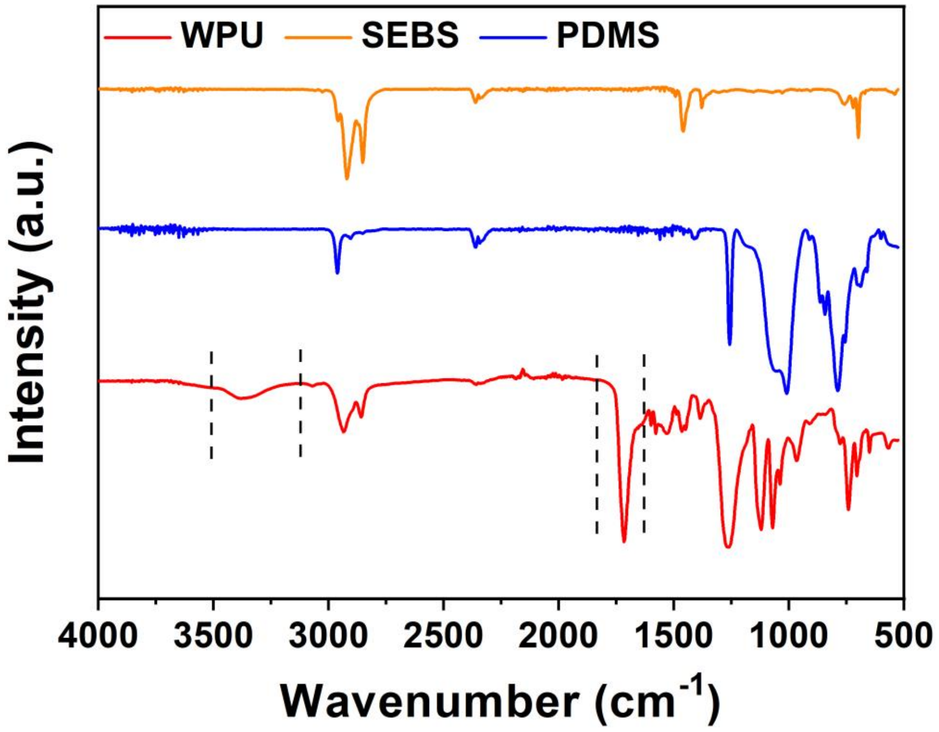

Supplementary Figure 2 Fourier transform infrared spectra (FTIR) of WPU, SEBS and PDMS film.

In the FTIR spectrum of WPU, the band at 3500 ~ 3200 cm^{-1} is attributed to –OH and –NH- stretching vibration, and the band at 1800 ~ 1600 cm^{-1} is attributed to carboxyl stretching vibration[1, 2]. This result proves that WPU molecular chain has hydrophilic functional groups. In contrast, FTIR spectra reveal that there are no hydrophilic polar groups in the molecular chains of PDMS and SEBS.

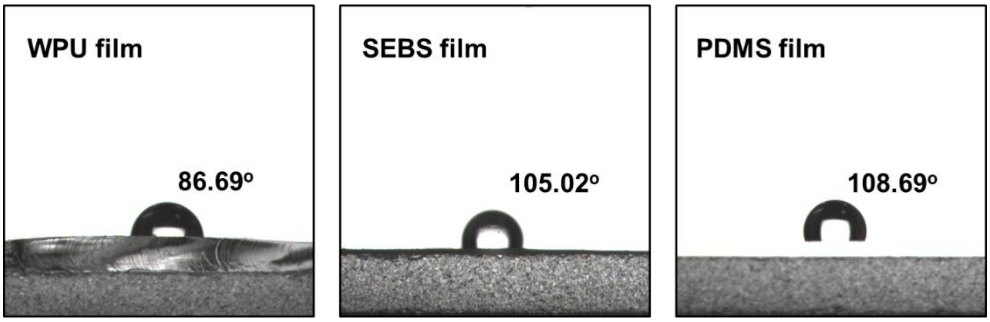

Supplementary Figure 3 Snapshots of a water droplet spreading on the WPU, SEBS and PDMS film.

When a water droplet contacted the WPU film surface, it spread and a water contact angle of 86.69^o was achieved, suggesting the hydrophilicity of the WPU film. In contrast, the water contact angle of SEBS and PDMS film is 105.02^o and 108.69^o respectively, indicating the hydrophobic property of those elastic films[3].

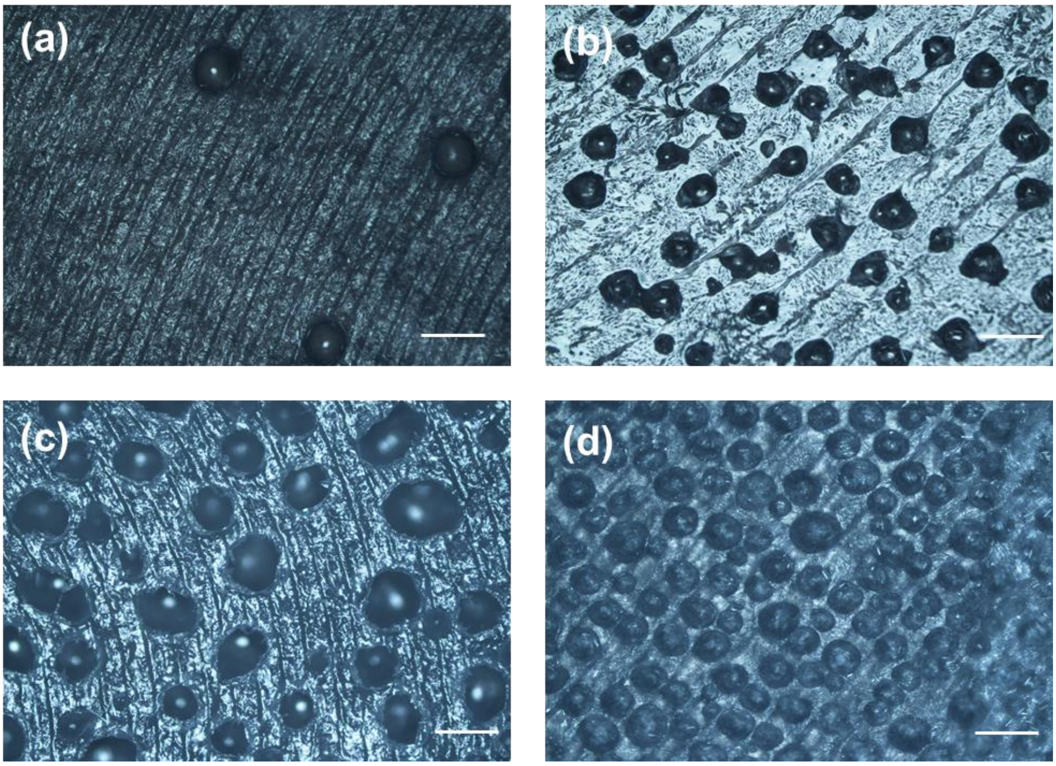

Supplementary Figure 4 Microscope images of WPU films as a function of urea content at (a) 5%, (b) 10%, (c) 15% and (d) 20%. Scale bar: 300 μm. Each experiment was repeated three times independently with similar results.

The WPU film with 5 wt% urea has sparse micropores. In addition, both WPU films with 10 wt% and 15 wt% urea show uniform permeable micropores. The pore size of the former is around 100 μm, while most of micropores in the latter are larger than 200 μm. The WPU film with 20 wt% urea obtains dense closed micropores that are not breathable.

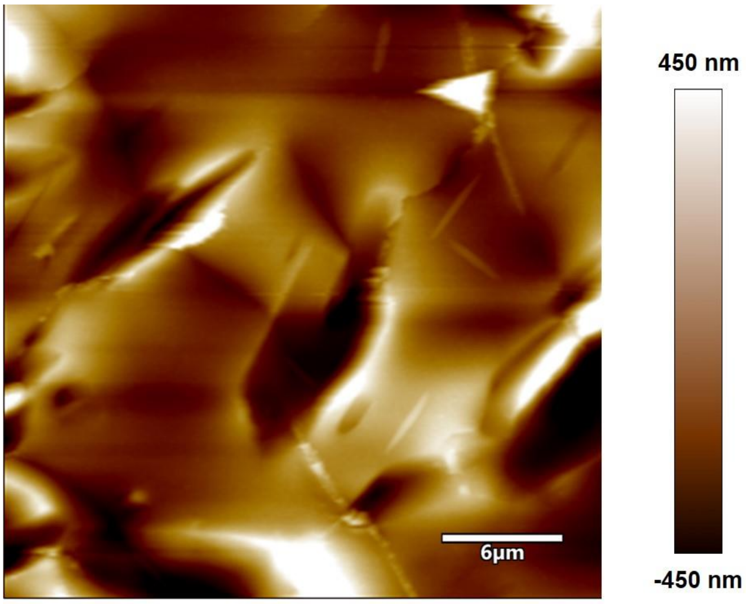

Supplementary Figure 5 AFM height distribution image of Au-RPU device. Scale bar: 6 μm. Each experiment was repeated three times independently with similar results.

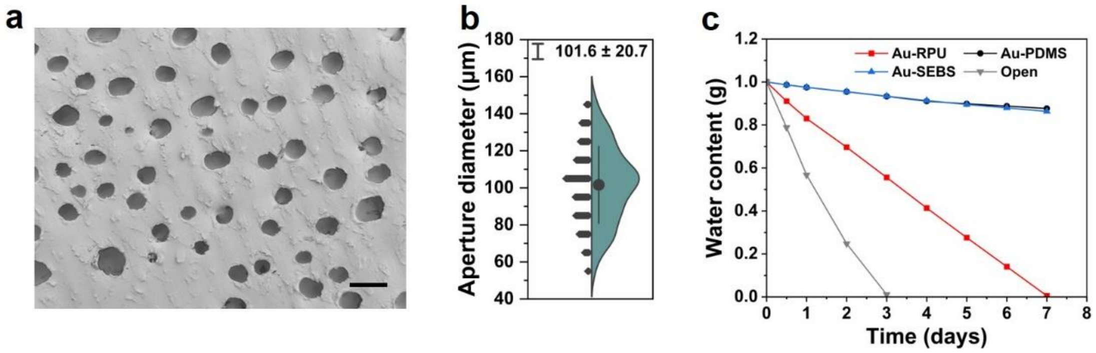

Supplementary Figure 6 (a) SEM image of Au-RPU device with rough microporous structure. Scale bar: 200 μm. Each experiment was repeated three times independently with similar results. (b) Pore diameter statistics of Au-RPU from SEM images. The p-value is 0.05. It indicates that the average pore diameter of Au-RPU device is 101.6 ± 20.7 μm (mean ± SD). (c) Water vapor permeability tests of Au-RPU, Au-SEBS and Au-PDMS on-skin electronics.

As for water vapor evaporation test, it was conducted by storing the samples in an incubator at 35 ^{o}C. The Au-RPU, Au-SEBS and Au-PDMS on-skin electronics were attached to the opening of a bottle containing 1 g deionized water, and the gas permeability was tested by measuring the weight loss of water. The water in the bottle with the Au-RPU device evaporated completely after one week, while the weight of the bottle barely decreased (87% of the water remain) over the same time. This result shows a high degree of gas permeability for our Au-RPU device.

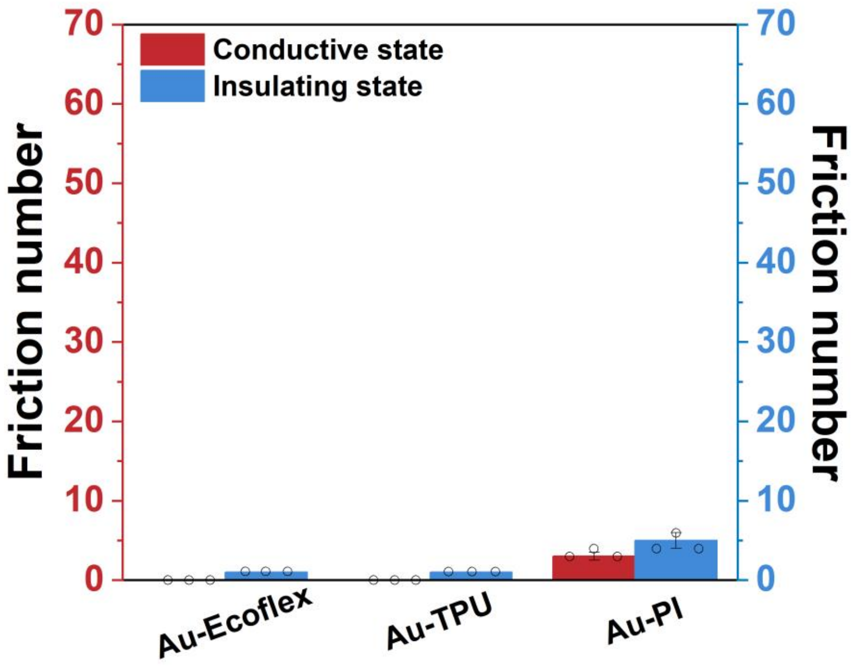

Supplementary Figure 7 Anti-friction performance of Au-Ecoflex, Au-TPU and Au-PI upon cyclic frictions using an artificial aging skin under 130 kPa pressure. Data are presented as mean values ± SD, n = 3 independent samples.

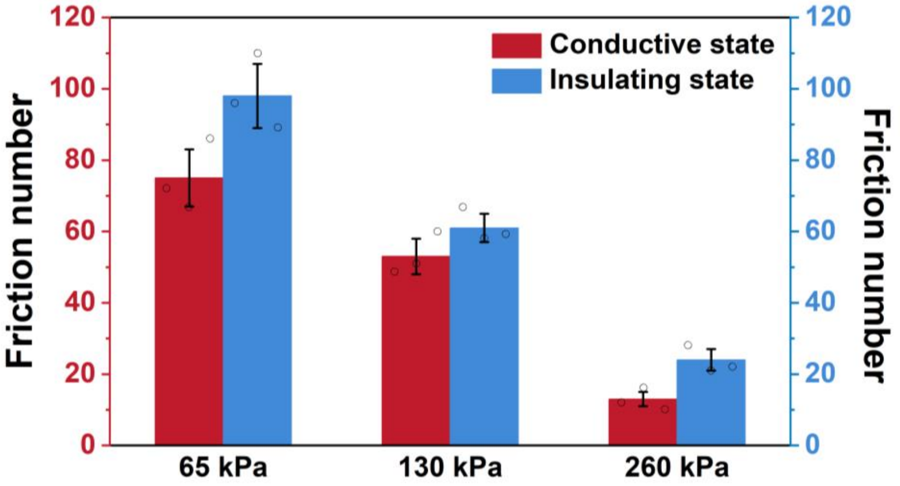

Supplementary Figure 8 Electrical performance of Au-RPU device under 65 kPa, 130 kPa and 260 kPa vertical pressure. The friction object is artificial aging skin. Data are presented as mean values ± SD, n = 3 independent samples.

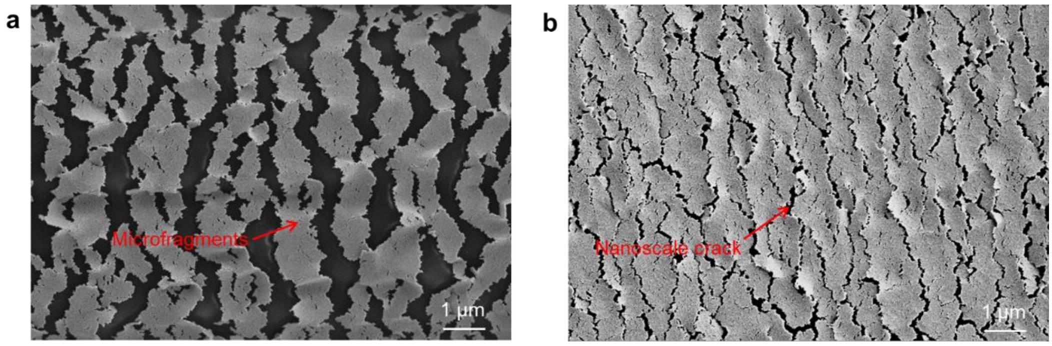

Supplementary Figure 9 SEM images of our Au-RPU device (a) during and (b) after 200% strain. Each experiment was repeated three times independently with similar results. Nanoscale cracks in the Au films are formed without obvious slippage due to the strong interfacial adheison.

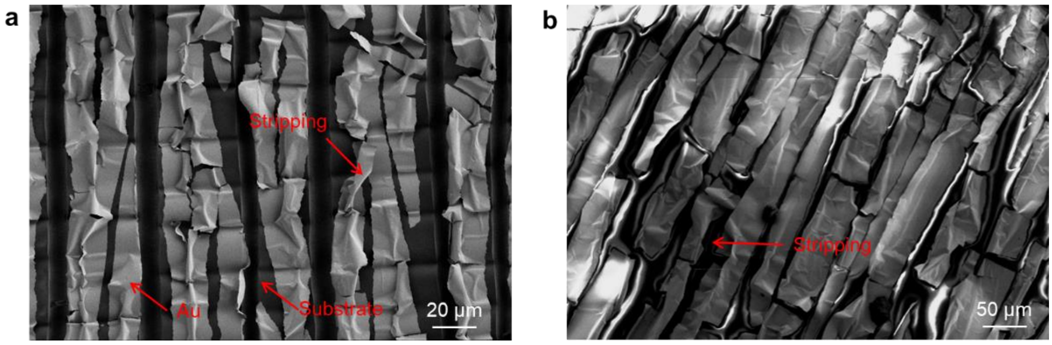

Supplementary Figure 10 SEM images of Au-PDMS device (a) during and (b) after 200% strain. Each experiment was repeated three times independently with similar results. Significant stripping in the Au films are observed during the tensile strain, resulting in disconnected large size fragments.

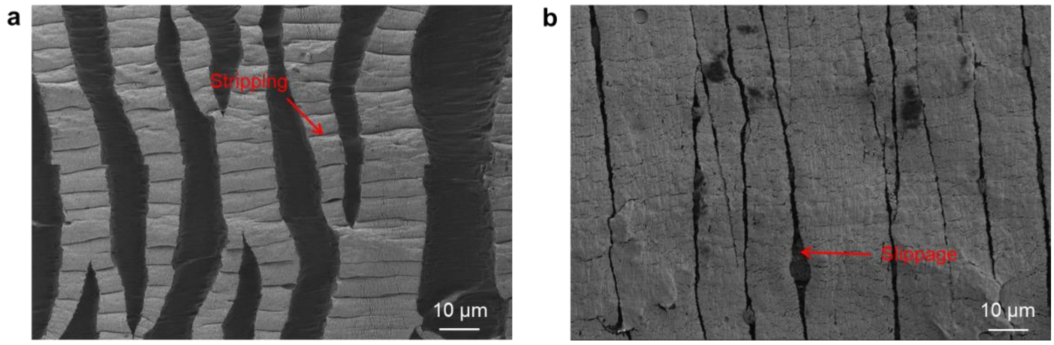

Supplementary Figure 11 SEM images of Au-SEBS device (a) during and (b) after 200% strain. Each experiment was repeated three times independently with similar results. Microcracks are observed at the released state, indicating the slippage of Au layer after the stretching. As a result, the electrical conductivity of the device decreases.

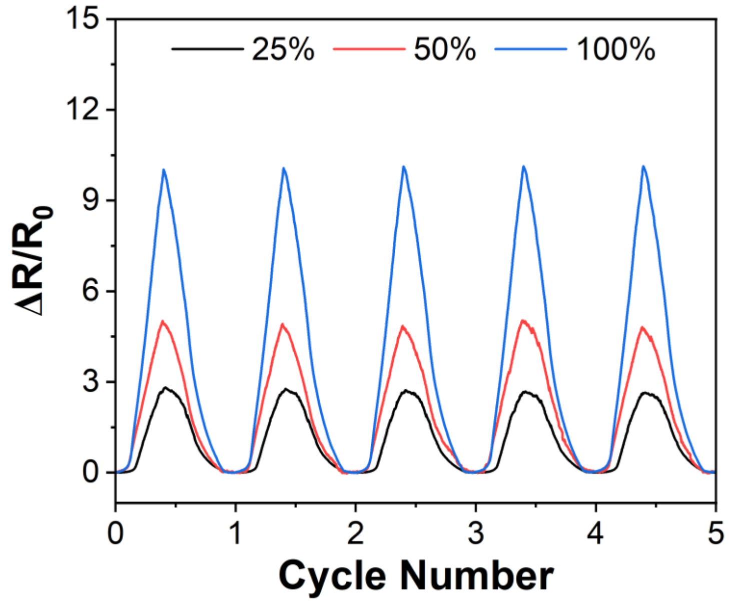

Supplementary Figure 12 Stability of the Au-RPU device against cyclic tensile deformation at 25%, 50% and 100% strain.

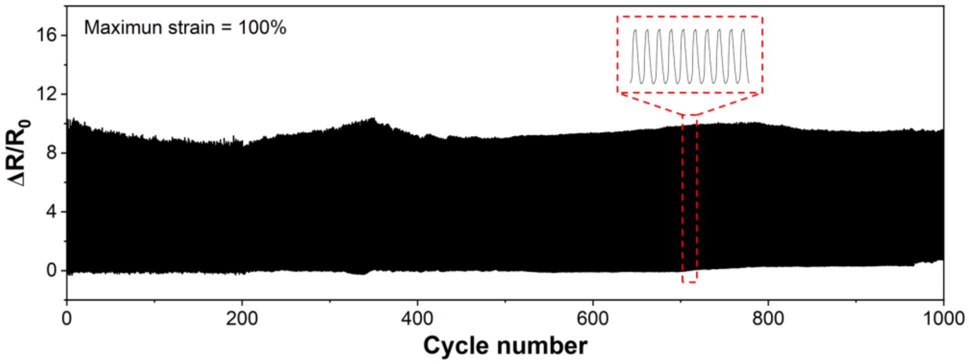

Supplementary Figure 13 Stability of the Au-RPU device during 1,000 cycles of the stretch/release process at 100% tensile strain. Inset shows the 10 cycles of the stretch/release process from 701 to 710 cycle.

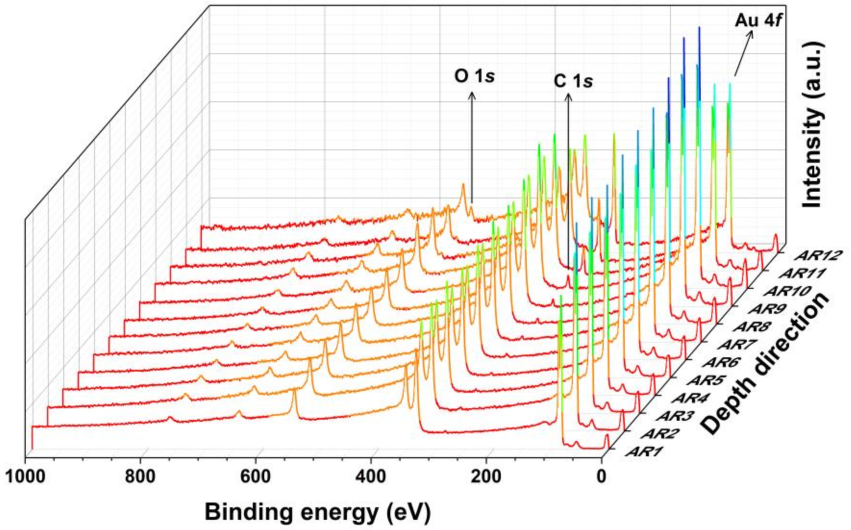

Supplementary Figure 14 XPS survey spectra of the 20 nm-thick Au layer on smooth PU substrate at different Ar^+ ion etching levels.

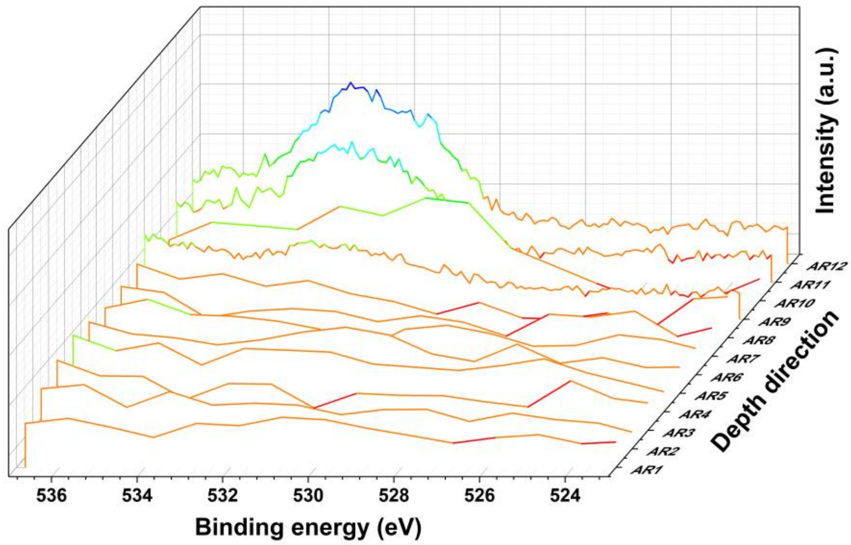

Supplementary Figure 15 O 1*s* XPS spectra of the 20 nm-thick Au layer on smooth PU substrate at different Ar^+ ion etching levels.

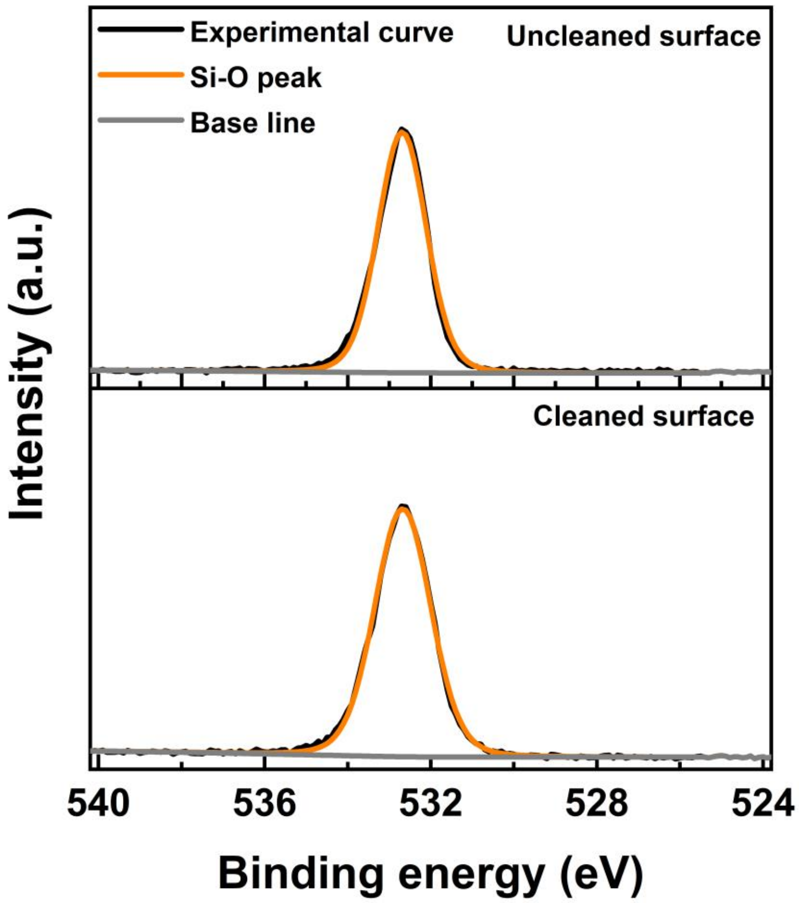

Supplementary Figure 16 O 1*s* XPS spectra of PDMS film before and after surface cleaning by Ar^+ ion.

The Si-O band at 532.8 eV is obtained and shows no obvious change before and after surface cleaning, indicating that no chemcial groups or water molecules are absorbed on PDMS surface.

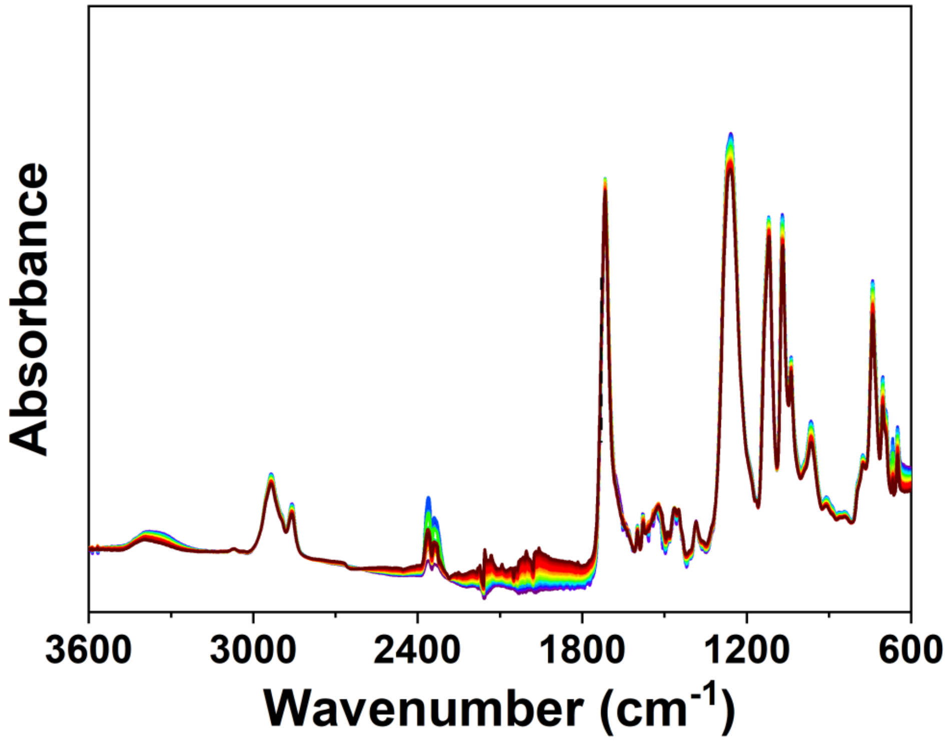

Supplementary Figure 17 Temperature-dependent FTIR spectra of WPU upon heating from 30 °C to 100 °C.

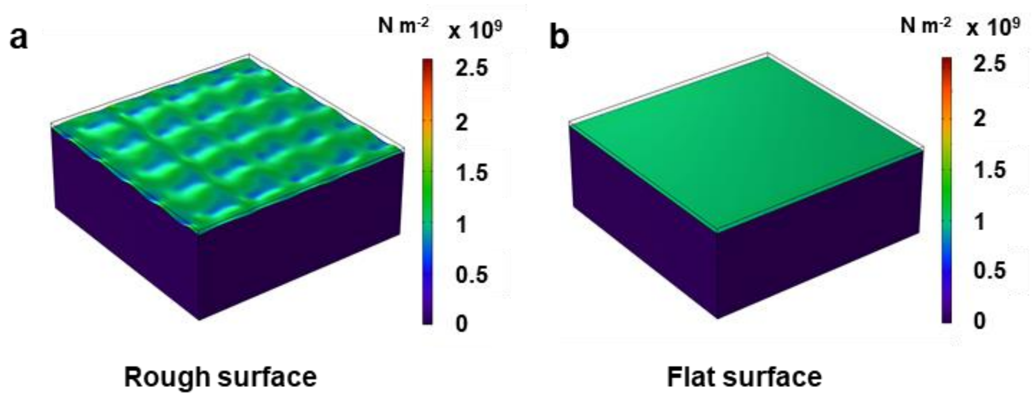

Supplementary Figure 18 Comparison of the simulated deformation stress distribution of the Au layer on (a) RPU and (b) flat WPU substrate, showing an enhanced interface binding strength of the Au-RPU device.

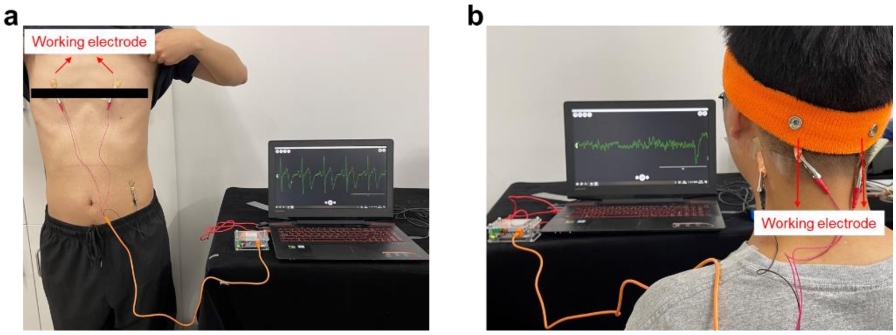

Supplementary Figure 19 Optical images of (a) ECG and (b) EEG detection on the male volunteer.

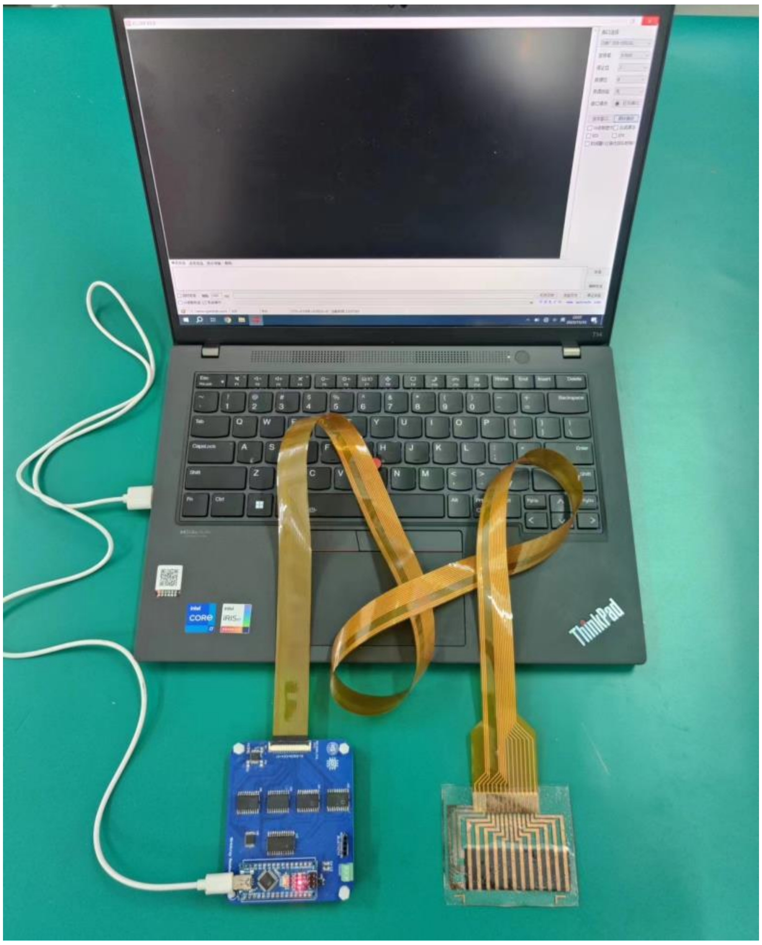

Supplementary Figure 20 Optical image of our 3 × 12 pressure sensor array based on Au-RPU electrode and the signal readout hardware circuit.

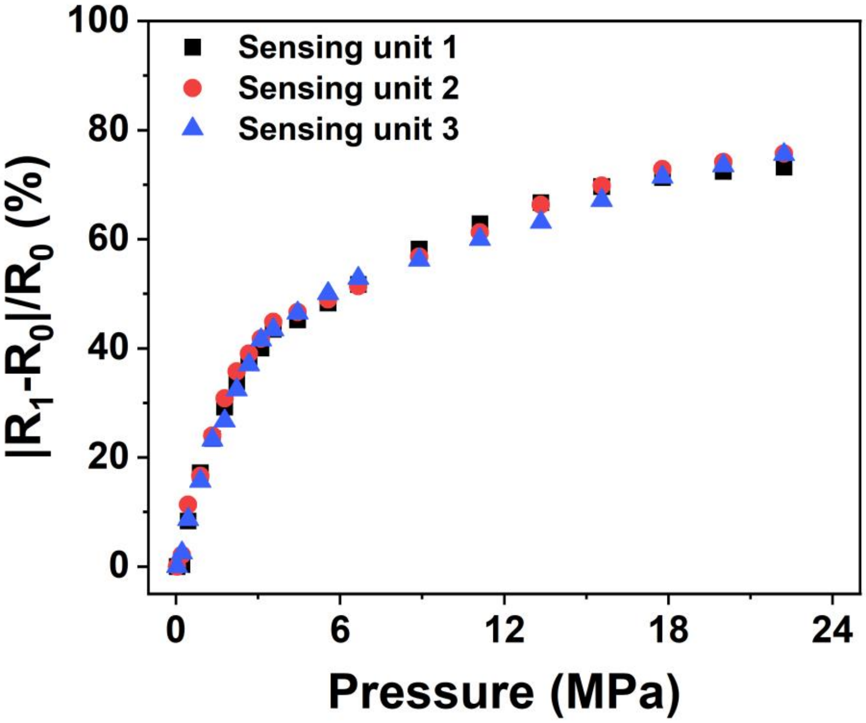

Supplementary Figure 21 Response of three separate sensors in the force range of 0 to 22.2 MPa.

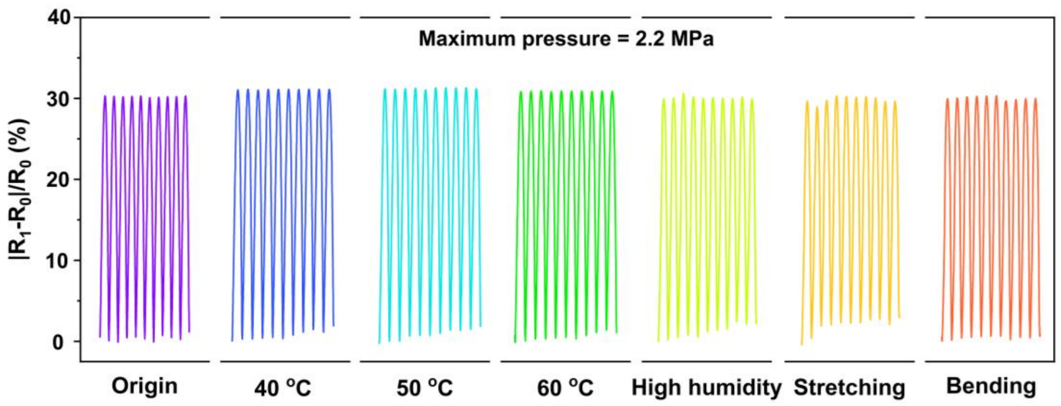

Supplementary Figure 22 Response of the pressure sensor during the cyclic loading/unloading process at 2.2 MPa pressure under various environmental conditions, including the original, 40 ^{o}C, 50 ^{o}C, 60 ^{o}C, high humidity, stretching and bending state.

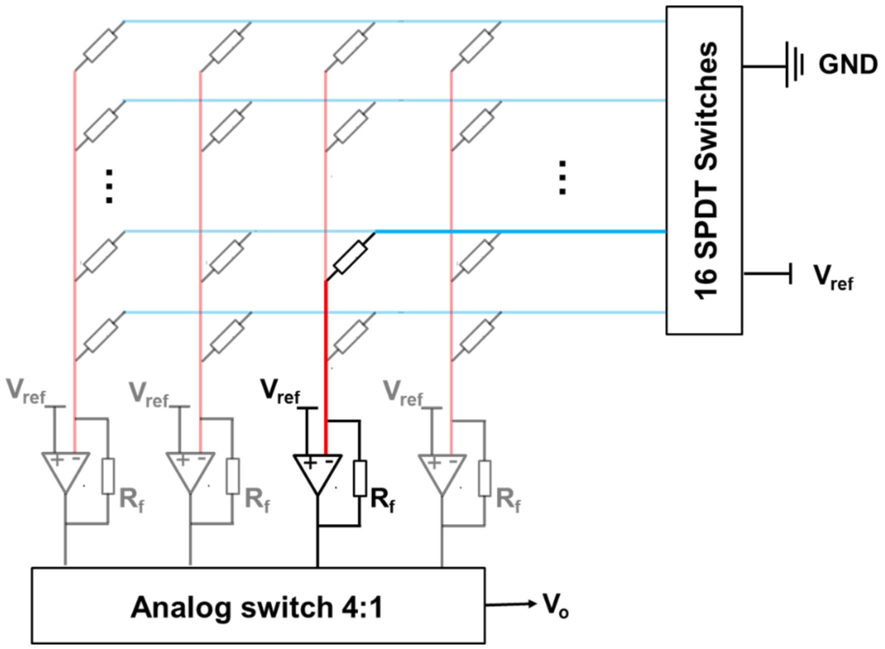

Supplementary Figure 23 Schematic diagram of the APSA readout circuit.

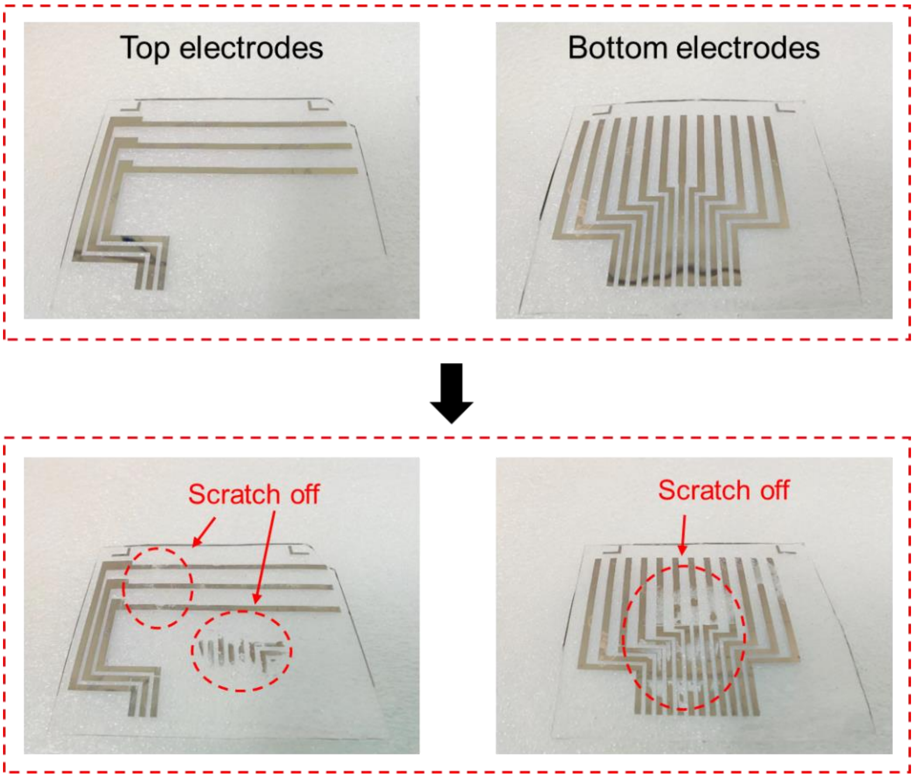

Supplementary Figure 24 Photograph showing the surface morphology of Au-PDMS top and bottom electrodes before and after device assembly. Most of the patterned Au layers were scratched off after the device was assembled and subjected to small pressing force or bending deformation.

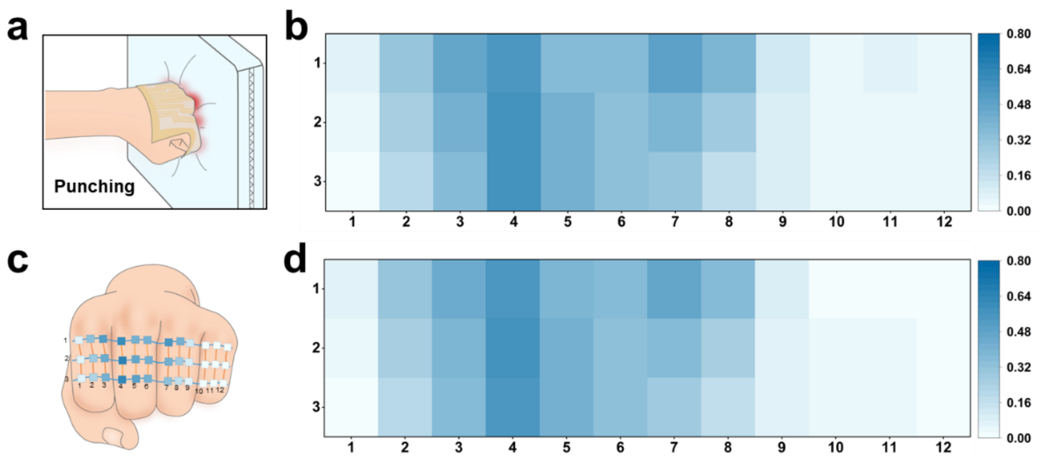

Supplementary Figure 25 (a) Schematic illustration of pressure sensor array attached on the fist to record the force when punching the baffle. (b) Pressure force maps of the original pressure sensor array when beating the baffle. (c) Schematic illustration of pressure force maps when beating the baffle. (d) Pressure force maps of the pressure sensor array after 1000 pressing deformations.

When the fist of a male volunteer wearing the sensor array beat a soft baffle vertically (Supplementary Fig. 25a), the obtained pressure mapping was shown in Supplementary Fig. 25b. The pressure pixels on the proximal phalangeal surface of the middle finger were the largest in the mapping, and gradually decreased toward the proximal phalangeal surface of the fingers on both sides. Notably, our device was able to reliably record the magnitude and distribution of the beat forces even after the Au electrode underwent 1000 cyclic interfacial frictions by 4.4 MPa vertical pressure (Supplementary Fig. 25c, d).

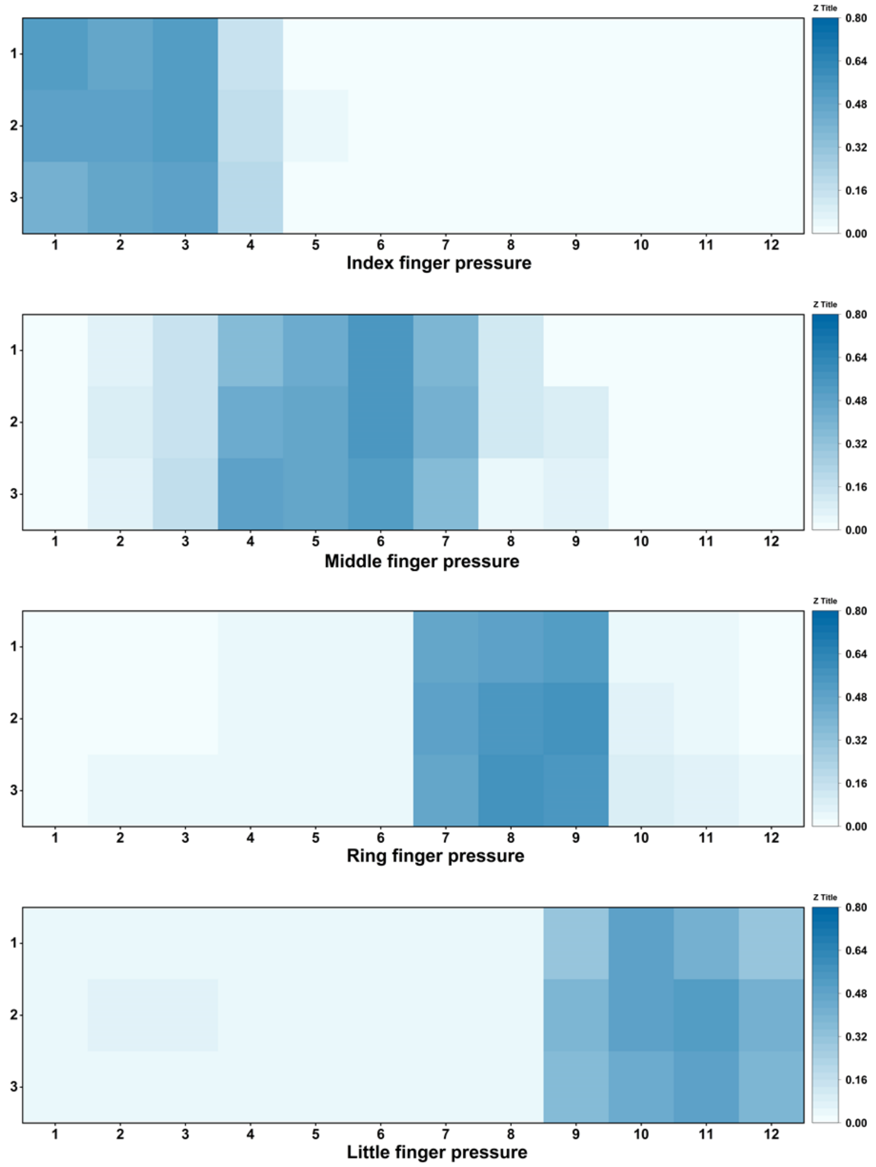

Supplementary Figure 26 Pressure force maps of the pressure sensor array when the fist's index finger, middle finger, ring finger and little finger beat the baffle, respectively.

It was amenable to achieve distinct pressure mappings by utilizing individual fingers such as the index, middle, ring and little finger to strike the baffle (Supplementary Fig. 25), corroborating the capability of our device to accurately acquire concentrated force signals.

II: Supplementary Tables

Supplementary Table 1 Young's modulus of different flexible substrate materials.

	WPU	PDMS	SEBS	ECOFLEX	TPU	PET	PI
Young's modulus (MPa)	1.28	2.16	5.77	0.47	25.15	2102.82	805.40

Supplementary Table 2 Comparison of the interfacial binding strength of our device with other reported works.

**Device structure**	**Interface design**	**Test method**	**Peel strength (N/m)**	**Reference**
Au/SEBS	Biphasic interpenetrating nanostructure	T-peel test	120 N/m	Nature 2023, 614, 456.
Au/PU fiber/PAM hydrogel	Mechanically interlocked interface	90° -peel test	< 60 N/m	Adv. Funct. Mater. 2020, 30, 1909540.
Au(Pt)-Ti/Polyimide	Chemical modification	T-peel test	< 400 N/m	Adv. Mater. Technol. 2021, 6, 2100149.
Pt-Ti/ Off-stoichiometry thiol-ene-epoxy film	Direct deposition	T-peel test	< 100 N/m	Biomaterials, 2023, 293: 121979.
Cu tape/Kapton film	Direct paste	T-peel test	~ 500 N/m	IEEE International Conference on Electro Information Technology, 2021, 409.
Au-RPU	Diffusion-induced cohesion	T-peel test	1243.4 N/m	**This work**
Au-WPU	Diffusion-induced cohesion	T-peel test	1017.6 N/m	**This work**

Supplementary Table 3 Comparison of electrical property and anti-friction performance of stretchable devices with different conductive materials.

**Device type**	**Stability in the air**		**Number of anti-friction (a.u.)**	
	**Conductivity after deposition**	**Conductivity in the air**	**Conductive state**	**Insulating state**
Au-RPU	Conductive	Permanently conductive	53 ± 5	61 ± 4
Pt-RPU	Conductive	Permanently conductive	49 ± 5	64 ± 3
Ag-RPU	Conductive	Non-conductive after 1 month	\	\
Cu-RPU	Conductive	Non-conductive after 3 hours	\	\
Conductive state: R < 500 Ω/□<br>Insulating state: R > 100 MΩ/□				

Supplementary Table 4 Surface roughness of different friction objects.

**Friction objects**	**Surface roughness**	
	**Ra**	**Rq**
Abrasive paper	2.44 μm	3.14 μm
Woven fabric	1.10 μm	1.29 μm
Aging skin	445.76 nm	618.44 nm
Tender skin	5.83 nm	7.86 nm

Supplementary Table 5 Comparison of electrical properties and anti-friction ability of gold-based stretchable electronics.

Device	Conductive mechanism	Electrical property upon strain	Anti-friction ability	Reference
Au-PU	Layer-by-layer assembly of Au	1×10^6 S/m, 0% strain; Break, 120% strain	N/A	Nature 2013, 500, 59-63.
	Au-PU blending	1×10^5 S/m, 0% strain; 5×10^3 S/m, 400% strain		
Au-PVA nanomesh	Microcracks	3×10^{-3} S, 0% strain; Break, ~50% strain	N/A	Nat. Nanotech. 2017, 12, 907.
Au-Supramolecular polymeric materials	Microcracks	Low resistance, 0% strain; Break, ~400% strain	N/A	J. Am. Chem. Soc. 2018, 140, 5280.
Au-Parylene-Polyurethane	Microcracks	55 Ω, 0% strain; Break, ~40% strain	N/A	Nat. Nanotech. 2019, 14, 156.
Au-PDMS	Microcracks	25-300 Ω, 0% strain; Break, ~70% strain	N/A	Adv. Mater. 2016, 28, 6359.
Au-PDMS meshed film	Microcracks	12 Ω/sq, 0% strain; Break, 94% strain	N/A	ACS Sens. 2020, 5, 3165.
Au-Shape memory polymer	Microcracks	85 Ω/sq, 0% strain; Break, ~200% strain	N/A	Appl. Phys. Lett. 2016; 108, 061901.
Au-PDMS	Microcracks	500 Ω, 0% strain; Break, ~120% strain	N/A	J. Appl. Phys. 2019, 125, 165305.
Au-PDMS	Microcracks	~20 Ω, 0% strain; ~1 kΩ, 300% strain; Break strain, not given	N/A	Nat. Electron. 2022, 5, 784.
Au-PVA-PU	Microcracks	Pressure sensor: $\Delta C/C_0 = 1.4$, 100 kPa pressing.	Force: 0.5 N;<br><br>Encapsulation: polyurethane layer;<br><br>Performance: remain stable at provided 300 frictions.	Science 2020, 370, 966.

Au-RPU	Microcracks	~3 × 10^6 S/m, 0% strain;<br>~ 1 × 10^3 S/m, 400% strain	Force: 10 N (130 kPa);<br><br>No encapsulation;<br><br>Performance: Remain conductivity after 1022 ± 76 frictions.	**This work**

Supplementary Table 6 Atomic relative concentration of C 1s, O 1s and Au 4f from XPS depth profiling of the 20 nm-thick Au layer on WPU substrate.

Atomic relative concentration（%）			
	C 1s	O 1s	Au 4f
AR1	6.49	\	93.51
AR2	4.79	\	95.21
AR3	6.18	\	93.82
AR4	6.89	\	93.11
AR5	11.98	\	88.02
AR6	12.47	\	87.53
AR7	19.09	\	80.91
AR8	25.9	\	74.1
AR9	36.95	**2.08**	60.96
AR10	57.06	**3.04**	39.9
AR11	79.68	**4.01**	16.31
AR12	86.43	**4.85**	8.72

Supplementary Table 7 Relative weight content of three fitting peaks for O 1s high resolution XPS spectra.

	O_I(O-H peak)		O_{II}(-COO- peak)		O_{III}(C=O peak)	
	Position (eV)	%Area	Position (eV)	%Area	Position (eV)	%Area
AR 12	533.38	**44.35**	532.02	36.36	530.84	19.27
Uncleaned WPU	533.37	**50.50**	531.98	40.76	531.22	8.74
Cleaned WPU	533.34	**39.46**	532.29	48.24	531.03	12.30

Supplementary Table 8 Comparison of our pressure sensor array with the metal-electrode-based pressure sensors.

**Device electrode**	**Electode stretchability**	**Sensing performance**			**Anti-friction ability**	**Reference**
		**Sensitivity**	**Detection range**	**Long-term stability**		
Ag nanowire electrode	No	$\Delta I/I_0 = 17$, $0 \leqslant P \leqslant 1$ kPa; $\Delta I/I_0 = 10$, $2 \leqslant P \leqslant 10$ kPa	0-10 kPa	Yes	N/A	ACS Nano 2022, 16, 368.
Ag nanoparticle electrode	No	$\Delta I/I_0 > 100$, $0 \leqslant P \leqslant 100$ kPa	0.05-900 kPa	Yes	N/A	Adv. Mater. Technol. 2022, 7, 2100428.
Ag nanoparticle electrode	No	$\Delta I/I_0 \approx 50$, $0 \leqslant P \leqslant 50$ kPa	5-600 kPa	Yes	N/A	Adv. Mater. Interfaces 2022, 9, 2200621.
Ag paste interdigital electrode	No	$\Delta I/I_0 \approx 70$, $0 \leqslant P \leqslant 15$ kPa	0-667 kPa	Yes	N/A	Smart Mater. Struct. 2019, 28 105027.
Cu foil electrode	Yes	$\Delta R/R_0 \approx 60\%$, $0 \leqslant P \leqslant 1$ kPa	0-20 kPa	Yes	N/A	Composites Part B 2021, 225, 109243.
Cu wire electrode	No	$\Delta I/I_0 \approx 0.2$, $0 \leqslant P \leqslant 1$ kPa	0-20 kPa	Yes	N/A	J. Mater. Chem. C 2020, 8, 16774
Cr/Au electrode	No	$\Delta R/R_0 \approx 0.25\%$, $0 \leqslant P \leqslant 30$ kPa	0-30 kPa	Yes	N/A	Nat. Biomed. Eng. 2020, 4, 997.
Cr/Au bottle electrode<br><br>CNT top electrode	Yes	$\Delta R/R_0 \approx 99\%$, $0 \leqslant P \leqslant 2.5$ kPa; $\Delta R/R_0 \approx 90\%$, $2.5\ \text{kPa} < P \leqslant 90$ kPa	0-90 kPa	Yes	N/A	Science 2018, 360, 998.

Cr/Au electrode	No	$\Delta I/I_0 \approx 0.8$, $0 \leqslant P \leqslant 0.15$ kPa	0-50 kPa	N/A	N/A	Sensor. Actuat. A 2018, 280, 261.
Ti/Au electrode	No	$\Delta C/C_0 \approx 7$, 10.1 dBar $\leqslant P \leqslant$ 10.9 dBar	10.1-10.9 dBar	N/A	N/A	npj Flex. Electron. 2018, 2, 13.
Au electrode	Yes	0.141 kPa^{-1}, < 1 kPa; 0.01 kPa^{-1}, > 10 kPa 100 kPa pressing.	0-100 kPa	Yes	Anti-friction ability by polyurethane encapsulation layer	Science 2020, 370, 966.
Au electrode	Yes	$\Delta R/R_0 \approx 40\%$, $0 \leqslant P \leqslant 3.1$ MPa; $\Delta R/R_0 \approx 33\%$, 3.6 MPa $\leqslant P \leqslant$ 22.2 MPa	0-22.2 MPa	Yes	Excellent anti-friction ability due to DIC strategy	**This work**

III: Supplementary Note 1

XPS depth profile analyses are performed on 20-nm-thick Au/flat WPU (20-Au/WPU) sample. The contents change of Au 4*f*, O 1*s* and C 1*s* elements at the interface are analyzed by etching the Au layer with Ar ion gradually.

Notably, the XPS electron escape depth at 1486.7 eV for Au, O and C elements is around 1.7, 2.0 and 2.8 nm respectively, which means if O element is detected in Au film thicker than the oxygen escape depth (2.0 nm), then it can prove the diffusion of O element in Au layer. Since the total thickness of the Au layer is 20 nm and approximately 1.5 nm-thick Au layer can be removed per etching, the thickness of the Au layer after the ninth etching is around 6.5 nm. We detected O signal in Au layer at a depth of 6.5 nm. This depth is higher than the XPS electron escape depth of O element (2.0 nm). These results therefore confirm the diffusion of oxygen molecules or oxygen-containing groups into the Au layer.

Based on the FTIR spectrum of WPU, each O 1*s* XPS spectrum for surface uncleaned WPU or cleaned WPU is the convolution of three components: a -OH group signal at 533.3 ± 0.2 eV, a –COO- group signal at 532.1 ± 0.2 eV, and a C=O group signal at 531.0 ± 0.2 eV.

IV: Supplementary References

1. Cao J, *et al.* Arbitrarily 3D Configurable Hygroscopic Robots with a Covalent–Noncovalent Interpenetrating Network and Self-Healing Ability. *Adv. Mater.* **31**, 1900042 (2019).
2. Su G, Zhou T, Liu X, Zhang Y. Two-step volume phase transition mechanism of poly(N-vinylcaprolactam) hydrogel online-tracked by two-dimensional correlation spectroscopy. *Phys. Chem. Chem. Phys.* **19**, 27221-27232 (2017).
3. Law K-Y. Definitions for Hydrophilicity, Hydrophobicity, and Superhydrophobicity: Getting the Basics Right. *J. Phys. Chem. Lett.* **5**, 686-688 (2014).
