# Supplementary material for: Anti-friction gold-based stretchable electronics enabled by interfacial diffusion-induced cohesion
Source: Nat Commun. 2024 Feb 6;15:1116. doi: 10.1038/s41467-024-45393-x (PMC10847152; doi:10.1038/s41467-024-45393-x)
Supplement: Supplementary file 3 — Description of Additional Supplementary Files [file 41467_2024_45393_MOESM3_ESM.pdf]

Description of Additional Supplementary Files

Supplementary Movie Legends

Supplementary Movie 1: Electrical anti-friction performance comparison of the Au-WPU, AuSEBS and Au-PDMS device.

Supplementary Movie 2: Circuit setup for realtime ECG recording.

Supplementary Movie 3: ECG recording by antifriction Au-RPU electrodes and Au-PDMS electrodes.

Supplementary Movie 4: EEG recording by antifriction Au-RPU electrodes and Au-PDMS electrodes.

Supplementary Movie 5: Robust LED circuit under multiple finger-rubbing.

Supplementary Movie 6: Robust LED circuit under bending, kneading and stretching.

Supplementary Movie 7: Demonstration of APSA attached on the palm for pressure recording when grasping an apple.

Supplementary Movie 8: Demonstration of APSA attached on the palm for pressure recording when grasping a horned melon.

Supplementary Movie 9: Demonstration of APSA for cyclic grasping a horned melon.

Supplementary Movie 10: Assembly process of the pressure sensor array based on Au-PDMS electrodes.

Supplementary Movie 11: Demonstration of APSA used for force recording when punching.
